# Immunomodulatory Effects of Anesthetic Techniques in Lung Cancer Surgery: A Systematic Review and Meta-Analysis

**DOI:** 10.3390/medicina61071263

**Published:** 2025-07-12

**Authors:** Georgios Konstantis, Ilias Katsadouros, Georgia Tsaousi, Vasileios Grosomanidis, Chryssa Pourzitaki

**Affiliations:** 1 Laboratory of Clinical Pharmacology, School of Medicine, Aristotle University of Thessaloniki, 54124 Thessaloniki, Greecehliask77@gmail.com (I.K.); 2 Department of Gastroenterology, Hepatology and Transplant Medicine, Medical Faculty, University of Duisburg-Essen, 45141 Essen, Germany; 3 Department of Anesthesiology and ICU, School of Medicine, Aristotle University of Thessaloniki, 54124 Thessaloniki, Greece; tsaousig@otenet.gr (G.T.); vgrosoma@auth.gr (V.G.)

**Keywords:** lung cancer, surgery, anesthesia, immonumodulation

## Abstract

*Background and Objectives*: Lung cancer represents one of the principal causes of cancer-associated mortality worldwide. Despite the numerous novel therapeutic agents, surgical resection remains, in many cases, the mainstay treatment. A growing body of evidence indicates that the anesthetic technique of choice contributes to perioperative immunosuppression, thus having an impact on cancer recurrence and prognosis. The aim of this systematic review is to provide a thorough summary of the current literature regarding the modulation of the immune response induced by the various anesthetic techniques that are used in lung cancer surgery, with a particular emphasis on cellular immunity. *Materials and Methods*: PubMed, Scopus, and the Cochrane databases were systematically searched from November 2023 up to March 2024 to identify randomized controlled trials (RCTs) that met the eligibility criteria. *Results*: A total of seven RCTs were included. Four of the RCTs compared the administration of general anesthesia alone versus general anesthesia combined with epidural anesthesia. The subsequent meta-analysis showed that the combination of general and epidural anesthesia exerted a positive impact on the cell counts of the CD3+ cells (SMD −0.42, 95% Cl −0.70 to −0.13 24 h postoperatively and SMD −0.86 95% Cl −1.48 to −0.23 72 h postoperatively), the CD4+ cells (SMD −0.41 95% Cl −0.69 to −0.12 at the end of surgery and SMD −0.56 95% Cl −0.85 to −0.27 72 h later), and the CD4+/CD8+ ratio (SMD −0.31 95% Cl −0.59 to −0.02 immediately after surgery, SMD −0.50 95% Cl −0.86 to −0.14 24 h postoperatively, and SMD −0.60 95% Cl −0.89 to −0.31 72 h later). The pooled results regarding CD8+ and NK cell counts were inconclusive. The remaining three studies compared volatile-based anesthesia with total intravenous anesthesia (TIVA). Due to disparities between these studies, qualitative analysis was inconclusive, whereas quantitative analysis was not feasible. *Conclusions*: The supplementation of general anesthesia with epidural anesthesia favorably impacts CD3+ and CD4+ cell counts, as well as the CD4+/CD8+ ratio. The present results and the effects of anesthetic technique on other immune cells must be consolidated with further high-quality studies.

## 1. Introduction

Lung cancer ranks among the most frequent types of malignancy and represents a leading cause of cancer-related mortality worldwide [1]. Despite the implementation of innovative therapeutic approaches, such as immune checkpoint inhibitors and antibody–drug conjugates, surgical resection of the tumor remains, in many cases, the treatment of choice, rendering anesthesia indispensable.

Immune dysregulation has gained considerable attention as a key perioperative factor involved in the process of cancer recurrence and metastasis. Along with surgery, anesthetic drugs and anesthetic techniques represent major perioperative factors that contribute to the suppression of the immune system [2,3]. Anesthesia can also directly affect cancer cell biology in addition to activating the hypothalamic–pituitary–adrenal axis (HPA) and the sympathetic nervous system (SNS), leading to the suppression of cell-mediated immunity and the release of immunosuppressive cytokines [4,5]. In vitro studies have demonstrated the enhanced proliferation and migration of cancer cells induced by volatile anesthetics [6,7] as well as their proapoptotic effect on T lymphocytes [8]. Furthermore, propofol has been shown to have anti-metastatic potential through the inhibition of proliferation [9], positively influencing cell-mediated immunity [10]. Likewise, the addition of regional anesthesia to general anesthesia mitigates the neuroendocrine response, which is associated with the immunosuppressive effects of surgical stress [11] and contributes to the preservation of the T helper 1/T helper 2 (Th1/Th2) balance [12].

Thus, anesthetic technique is a potentially modifiable prognostic factor. The current data are inconclusive, and no consensus has been reached for a preferred approach. No meta-analysis to date has addressed the immunomodulatory role of anesthesia during lung cancer surgery and the prognostic value of different anesthetic techniques. The aim of this meta-analysis is to summarize the existing data, with a special focus on the changes induced by anesthesia on the populations of the CD3+, CD4+, CD8+ T cells, NK cells, and the CD4+/CD8+ ratio. Moreover, the present analysis aims to provide an evaluation of the impact that the choice of anesthetic technique has on the immune response against lung cancer. The question of the choice of anesthetic technique, with respect to its immunomodulatory aspects, in the clinical setting of lung cancer surgery remains unsettled.

## 2. Materials and Methods

This systematic review and meta-analysis were conducted in accordance with the principles of the PRISMA statement, as it thoroughly stated in the Supplementary Materials. We aimed to identify the eligible randomized controlled trials (RCTs) through a systematic literature search from November 2023 to March 2024 employing the electronic databases PubMed, Scopus, and the Cochrane Library. The relevant records were imported into EndNote 19, and any duplicate entries were eliminated. Two reviewers (GK and DK) independently screened titles and abstracts. Eligible articles were then reviewed in full. Any disagreements during study selection were resolved by a third reviewer (CP). Data were extracted in a standardized format, including publication year, follow-up duration, country, study design, patient demographics (age, sex), tumor type and stage, anesthesia modality and duration, surgical technique, and both primary and secondary endpoints.

### 2.1. Selection Criteria

Trials that explored the effect of the various anesthetic techniques during lung cancer surgery on subpopulations of T lymphocytes and NK cells were included in this meta-analysis. In the current study, the immunomodulatory role of general anesthesia, independently or in combination with epidural anesthesia, was investigated. General anesthesia included the administration of propofol, sevoflurane, or desflurane. Full-text RCTs were considered eligible for this systematic review and meta-analysis if the study population consisted of adult participants, compared the use of general anesthesia alone or in conjunction with epidural anesthesia for lung cancer surgery, or compared the use of volatile-based anesthesia with total intravenous anesthesia (TIVA). Trials that reported the implementation of different anesthetic technique combinations than those described were omitted. Surgical technique and the chosen analgesia protocol were not considered as reasons for excluding studies. Furthermore, cancer stage, concurrent medical condition, or length of hospital stay did not constitute exclusion criteria. In order to avoid systematic publication error, language restrictions were not imposed either as an eligibility criterion or in our search strategy.

### 2.2. Outcome Measurements

The outcomes assessed the impact of anesthesia techniques on immune cells through the comparison of CD3+, CD4+, CD8+, and NK cell counts and the CD4+/CD8+ ratio at the end of surgery, 24 h postoperatively, and 72 h postoperatively. An available case analysis was applied.

### 2.3. Data Collection and Extraction Process

The records resulting from this search were imported into a reference management software. Initially, any duplicate records were removed, and the articles and abstracts of the remaining articles were assessed by two independent reviewers (GK and IK) based on the inclusion criteria. Thereafter, the full text of the remaining eligible articles following initial screening was retrieved and assessed. The evaluation of a third reviewer (CP) resolved any discrepancies concerning study eligibility. Data regarding outcomes along with information about the country in which this study was conducted, participants’ age and gender, American Society of Anesthesiologists (ASA) physical status classification system, type and details about anesthesia and anesthetic techniques, the administration of postoperative analgesia, lung tumor types and staging, type of surgery, and trial characteristics were extracted into a spreadsheet with a predetermined format. WebPlotDigitizer (version 4.7) was employed to extract data from plots when required.

### 2.4. Quality Evaluation

The Cochrane Risk of Bias 2 tool (RoB 2) was utilized for quality assessment of the outcomes. The appraisal of the trials was performed by two independent reviewers (GK and IK). In any cases of disagreement, the decision was made upon discussion with a third reviewer (CP). A trial was classified as high risk if at least one domain was judged as high risk, while the overall risk classification was declared as low when all five domains were acknowledged as low risk. Studies that do not fulfill the above criteria were characterized as having some concerns.

### 2.5. Statistical Analysis

Standardized Mean Difference (SMD) and 95% Confidence Intervals (CIs) were the measures of choice for reporting outcomes. Hedge’s g estimation method was applied in order to synthesize different scales of continuous data. The selected studies were solely RCTs, and the final values were employed for all analyses. The random effect model was implemented as the designated method of analysis to account for potential heterogeneity. Heterogeneity was evaluated with the Cochrane chi-square test, and the degree of heterogeneity was expressed with the I^2^ statistic. We performed our analysis presuming that participants who were not included in the primary analysis were due to protocol indiscipline, lost to follow-up, or other reasons, and were missing at random; available case analysis was used. All statistical analyses were performed with the Review Manager (RevMan) software (version 5.4).

## 3. Results

### 3.1. A Literature Search

**Figure 1:**
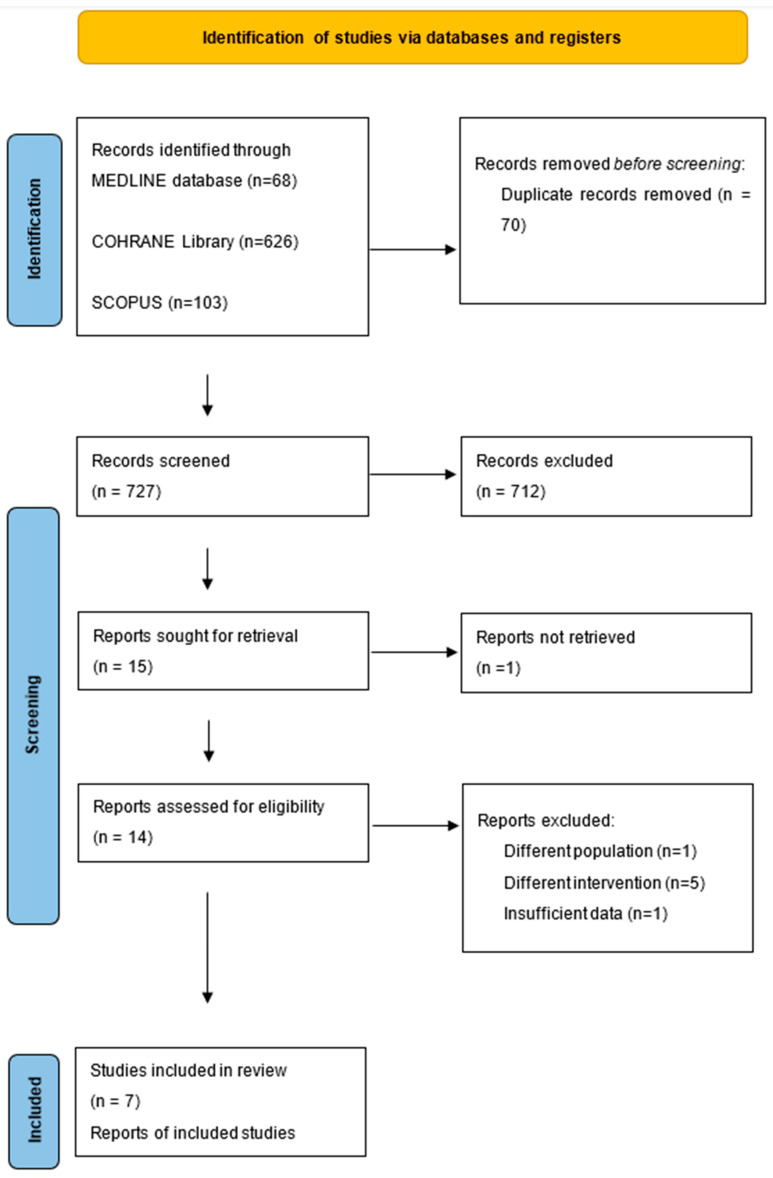
Prisma 2020 flow diagram.

The literature screening process is illustrated in Figure 1. Following the exclusion of irrelevant studies, seven RCTs [13,14,15,16,17,18,19] with a combined study population of 668 patients were eligible and subsequently included in this systematic review. Three RCTs [13,16,17] encompassing 346 patients compared the impact of volatile-based anesthesia versus total intravenous anesthesia (TIVA) on the immune response following lung cancer surgery. Four RCTs [14,15,18,19], including 322 patients, examined the effect of general anesthesia alone versus the administration of general anesthesia in conjunction with epidural anesthesia. Meta-analysis was feasible for 3 of the aforementioned RCTs, comparing general anesthesia versus general plus epidural anesthesia, with a total of 194 patients. Basic characteristics of the trials and the study subjects are outlined in Table 1.

### 3.2. Baseline Characteristics

Four RCTs explored the administration of standalone general anesthesia versus general anesthesia plus epidural anesthesia. In two studies [14,15], the lung cancer resection was performed through thoracoscopic surgery with either pneumonectomy, lobectomy, or a less complex approach, depending on the site of the tumor and its extent, while in the study of Zhu Y. et al. [18], patients underwent thoracotomy. Furthermore, Zhu Y. et al. [18] and Chen J. et al. [19] investigated the addition of epidural anesthesia to general anesthesia. Through the epidural catheter, ropivacaine was administered in the respective groups [14,18,19].

Three RCTs compared the use of volatile anesthetics with total intravenous anesthesia for patients undergoing lung cancer surgery. Two of them [13,17] evaluated TIVA versus sevoflurane as inhalational anesthesia. A third study [16] examined the administration of TIVA versus either sevoflurane or desflurane. In the studies of Yuan X. et al. [17] and Yamaguchi A. [16], the surgical method of choice was thoracotomy. In the Cui C. et al. study [13], the part of the lung resected was analogous to the extent of the disease.

The subjects of the aforementioned studies were diagnosed with various subtypes of lung cancer, staged 0 to IV TNM staging. Table 2 illustrates the features regarding intraoperative anesthesia.

### 3.3. Risk of Bias Assessment

Two independent investigators (GK and IK) utilized the RoB 2 tool to assess the risk of systematic error in the included studies. All seven studies were categorized as having “some concerns”, mainly due to either missing information regarding randomization or having issues with adherence to the intended intervention and the analysis of the effect of assignment. Examination of publication bias was not feasible due to the small number of eligible trials. Table 3 summarizes the results. Examination of publication bias was not feasible due to the small number of eligible trials.

### 3.4. Analysis of Outcomes

#### 3.4.1. General Anesthesia Versus General Anesthesia Plus Epidural Anesthesia

- CD3+ cell counts

**Figure 2:**
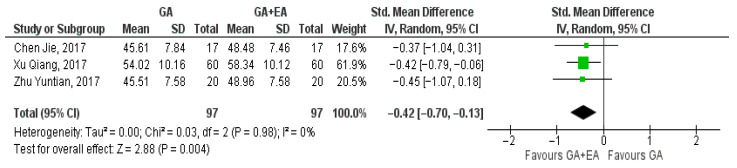
CD3+ cell count 24 h postoperatively [15,18,19].

**Figure 3:**
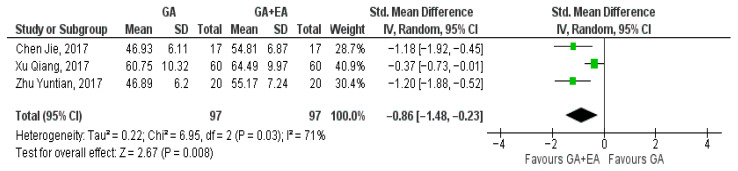
CD3+ cell count 72 h postoperatively [15,18,19].

**Figure 4:**
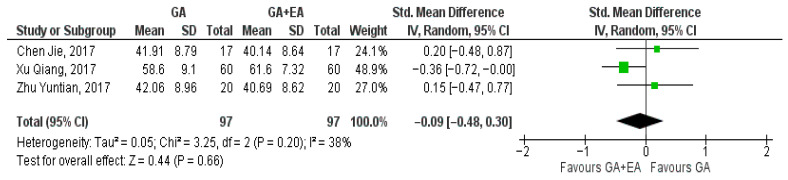
CD3+ cell count at the end of surgery [15,18,19].

Three studies [15,18,19] examined the effect of general versus general anesthesia plus epidural anesthesia on the number of CD3+ cells. Relevant data referring to the CD3+ counts were utilized to perform a comparison at the end of surgery, 24 h postoperatively, and 72 h postoperatively. Meta-analysis revealed a statistically significant elevation of the CD3+ cell count 24 h postoperatively (SMD −0.42, 95% Cl—0.70 to −0.13, I^2^ = 0%) (Figure 2) and 72 h postoperatively (SMD −0.86, 95% Cl −1.48 to −0.23, I^2^ = 71%) (Figure 3). CD3+ cell count was also elevated immediately after surgery (SMD—0.09, 95% CI −0.49 to 0.30, I^2^ = 38%), but no statistical significance was achieved (Figure 4).

b. CD4+ cell counts

**Figure 5:**
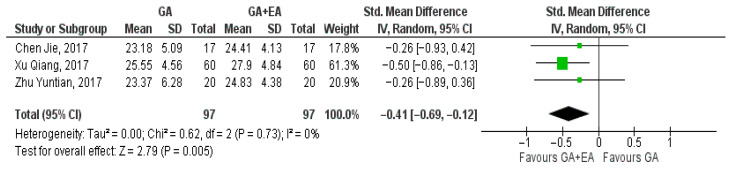
CD4+ cell count at the end of surgery [15,18,19].

**Figure 6:**
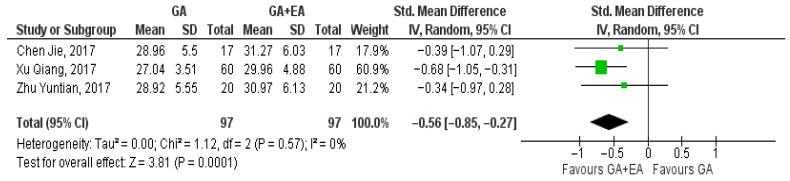
CD4+ cell count 72 h postoperatively [15,18,19].

**Figure 7:**
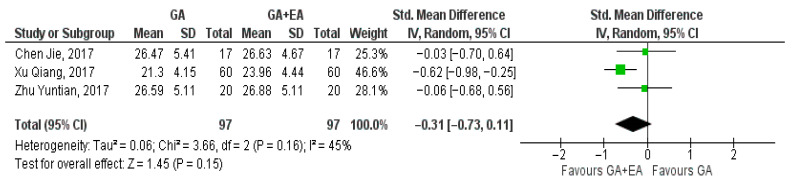
CD4+ cell count 24 h postoperatively [15,18,19].

Data extracted from three studies [15,18,19] comparing general anesthesia alone versus general anesthesia plus epidural anesthesia, regarding the number of CD4+ cells at the end of surgery, 24 h postoperatively, and 72 h postoperatively, were utilized for the meta-analysis. Data relevant to measurements of the CD4+ cell counts at the end of surgery, 24 h after the operation, and 72 h later were pooled. Our analysis illustrated that CD4+ cell counts were higher at the end of surgery (SMD −0.41, 95% CI −0.69 to −0.12, I^2^ = 0%) (Figure 5) and 72 h postoperatively (SMD −0.56, 95% CI −0.85 to −0.27, I^2^ = 0%) (Figure 6), achieving statistical significance. However, at the 24 h time point, CD4+ cell numbers were not significantly increased (SMD −0.31, 95% CI −0.73 to 0.11, I^2^ = 45%) (Figure 7).

c. CD8+ cell counts

**Figure 8:**
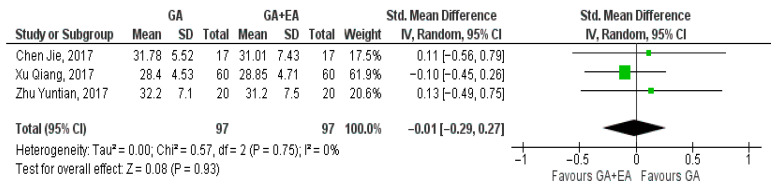
CD8+ cell count at the end of surgery [15,18,19].

**Figure 9:**
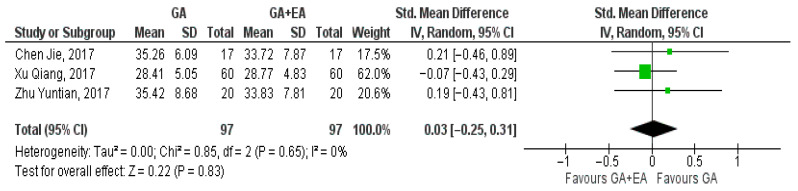
CD8+ cell count 24 h postoperatively [15,18,19].

**Figure 10:**
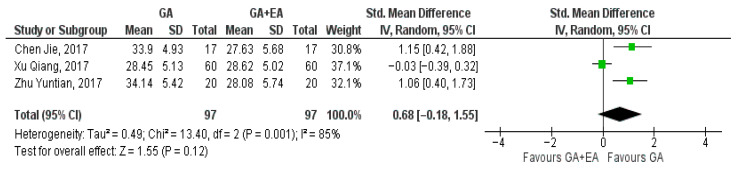
CD8+ cell count 72 h postoperatively [15,18,19].

The results of three studies [15,18,19] comparing general anesthesia with general plus epidural anesthesia were pooled at the end of surgery, 24 h later, and 72 h postoperatively. At the end of the surgery, the pooled result showcased a marginal increase in the CD8+ cell counts with general plus epidural anesthesia (SMD −0.01, 95% CI −0.29 to 0.27, I^2^ = 0%) (Figure 8) whereas 24 h later, a modest increase with general anesthesia was demonstrated (SMD 0.03, 95% CI −0.25 to 0.31, I^2^ = 0%) (Figure 9). Increased CD8+ cell numbers were illustrated 72 h postoperatively with the administration of general anesthesia but without reaching statistical significance (SMD 0.68, 95% CI −0.18 to 1.55, I^2^ = 85%) (Figure 10). In contrast with the three aforementioned studies that measured CD8+ cell counts in blood samples, Li, M.-H. et al. [14] measured the number of tumor-infiltrating CD8+ T cells. The number of CD8+ T cells per mm2 tumor was found to be higher in the general anesthesia plus epidural anesthesia group (median: 292.8 [interquartile range (IQR) 198.0, 418.3]) than that in the general anesthesia group (median 204.7 [IQR 131.1, 305.8]; *p* = 0.036).

d. CD4+/CD8+ ratio

**Figure 11:**
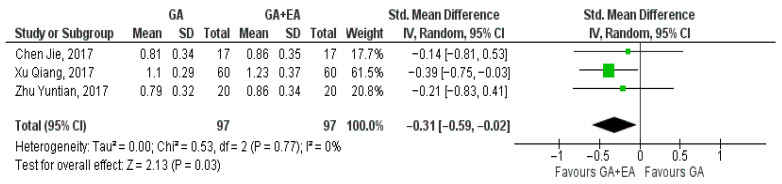
CD4+/CD8+ ratio at the end of surgery [15,18,19].

**Figure 12:**
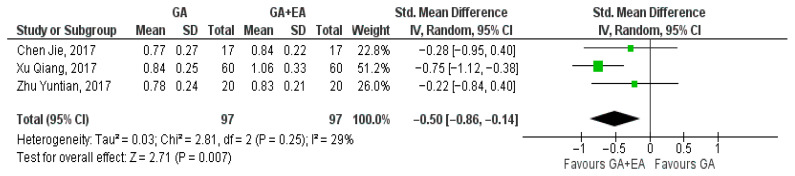
CD4+/CD8+ ratio 24 h postoperatively [15,18,19].

**Figure 13:**
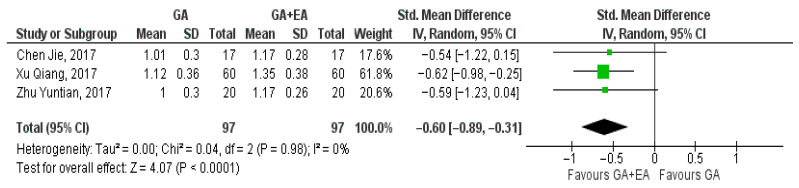
CD4+/CD8+ ratio 72 h postoperatively [15,18,19].

Data pertaining to the CD4+/CD8+ ratio were pooled from three studies [15,18,19]. A significant increase was shown with the administration of general anesthesia in conjunction with epidural anesthesia at the end of surgery (SMD −0.31, 95% CI −0.59 to −0.02, I^2^ = 0%) (Figure 11). Analysis at the 24 h time point revealed an additional increase in the CD4+/CD8+ ratio (SMD −0.50, 95% CI 0.86 to −0.14, I^2^ = 29%) (Figure 12). The ratio was further elevated 72 h postoperatively (SMD −0.60, 95% CI −0.89 to −0.31, I^2^ = 0%) Figure 13).

e. NK cell counts

**Figure 14:**
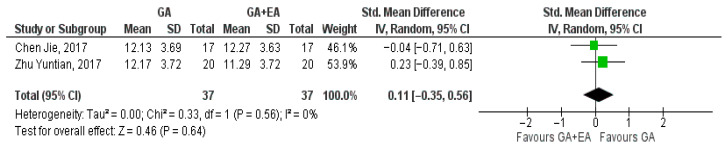
NK cell count at the end of surgerya [18,19].

**Figure 15:**
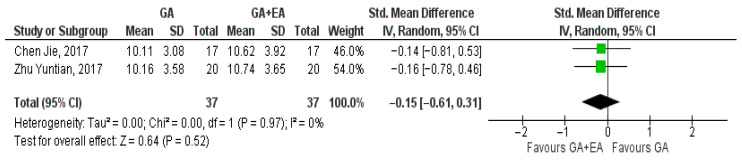
NK cell count 24 h postoperatively [18,19].

**Figure 16:**
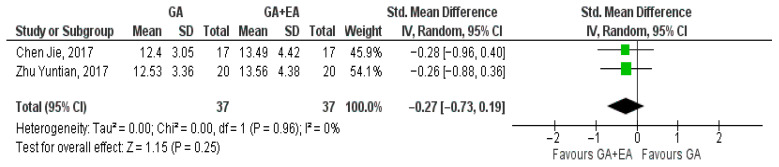
NK cell count 72 h postoperatively [18,19].

Two studies [18,19] evaluated the effect of different anesthetic techniques on the NK cell counts. Immediately after surgery, NK cell count was higher with the administration of general anesthesia (SMD 0.11, 95% CI −0.35 to 0.56, I^2^ = 0%) (Figure 14). Additionally, data pooled regarding 24 h (SMD −0.15, 95% CI −0.61 to 0.31, I^2^ = 0%) (Figure 15) and 72 h after surgery (SMD −0.27, 95% CI −0.73 to 0.19, I^2^ = 0%) (Figure 16) showed increased NK cell counts with the administration of combined general and epidural anesthesia. In all three cases, statistical significance was not detected.

#### 3.4.2. Volatile-Based Anesthesia Versus Propofol

- CD4+ cell counts

Yamaguchi A. et al. [16] in a three-armed study measured the impact of sevoflurane, desflurane, and propofol on CD4+ cell count at the end of surgery and found no difference. Yuan X. et al. [17] compared the administration of sevoflurane with propofol. The use of sevoflurane led to a statistically significant decrease in the number of CD4+ cells one day after the operation [mean ± SD: from 40.03 ± 0.87 to 5.85 ± 1.06 (pg/mL), *p* < 0.0001]. On the contrary, propofol administration significantly elevated the CD4+ cell count at the same time point [mean ± SD: from 39.10 ± 1.86 to 40.46 ± 0.93 (pg/mL), *p* < 0.0001]. Furthermore, the difference between the two groups was statistically significant (*p* < 0.0001). Due to the disparity between the time points at which the measurements were conducted, meta-analysis of the data was not feasible.

b. CD8+ cell counts

In the study of Yamaguchi A. et al. [16], the effect of sevoflurane, desflurane, and propofol on the number of CD8+ cells was examined. The administration of propofol elicited a decrease in the CD8+ cell count at the end of surgery [mean ± SD: +50.4 ± 23.2 to 37.5 ± 12.9 *p* < 0.05]. In the other groups, no respective decrease was observed. In the study of Yuan X. [17] the use of sevoflurane led to a lower number of CD8+ cells one day postoperatively [mean SD: from 26.39 ± 1.7 to 23.33 ± 1.364 (pg/mL), *p* < 0.0001], whereas an increase was showcased with the administration of propofol [mean SD: from 23.89 ± 1.97 to 26.99 ± 1.87 (pg/mL), *p* < 0.0001]. Additionally, statistical analysis between the two groups showed a significant difference in CD8+ cell count (*p* < 0.0001). A meta-analysis of the data was not possible as the blood samples from which the measurements were conducted were drawn at different time points.

c. CD4+/CD8+ ratio

In the study of Yuan X. [17], the administration of sevoflurane had nearly no effect on the CD4+/CD8+ ratio one day after surgery [mean ± SD: 1.52 ± 0.11 to 1.51 ± 0.13, *p* = 0.6217]. Conversely, with the use of propofol, a significant increase was observed [mean ± SD: 1.65 ± 0.15 to 1.51 ± 0.11, *p* =< 0.0001]. The comparison of the postoperative effect of the two anesthetic techniques revealed no significant difference (*p* = 0.7968).

d. T-reg cell counts

Yamaguchi A. et al. [16] evaluated the impact of desflurane, sevoflurane, and propofol on the proportion of regulatory T cells among T cells at the end of surgery. In the group of desflurane and that of propofol, no increase was observed. However, the administration of sevoflurane led to a significant increase in the percentage of Tregs in T cells. Yuan X. et al. [17] reported contradicting results, noting a statistically significant decrease with the use of sevoflurane in the number of T regulatory cells one day after surgery [mean SD: 1.81 ± 0.73 to 1.15 ± 0.360 (pg/mL), *p* < 0.0001]. This study additionally illustrated a significant increase in Treg cell count at the same time point with the administration of propofol [mean SD: from 1.25 ± 0.44 to 1.64 ± 0.7 (pg/mL), *p* < 0.0001]. The difference between the results of the sevoflurane and propofol groups was statistically significant (*p* < 0.0001). In another study, Cui C. et al. [13] compared the use of propofol with sevoflurane and measured the accumulation of regulatory T cells. One week after surgery, the propofol-treated patients had a significantly decreased accumulation of T regs compared to those in the sevoflurane group (2.78% vs. 3.27%).

## 4. Discussion

This systematic review and meta-analysis investigated the impact of different anesthetic techniques on immune regulation during lung cancer surgery. Particular attention was given to changes in NK cell counts and subsets of T lymphocytes. The findings suggest that general anesthesia combined with epidural anesthesia is associated with increased levels of CD3+ and CD4+ cells, as well as a higher CD4+/CD8+ ratio. However, pooled data regarding CD8+ and NK cell counts yielded inconclusive results. Due to methodological differences across the included studies, quantitative analysis was feasible only for comparisons between general anesthesia alone and general anesthesia with epidural anesthesia. Other studies comparing volatile anesthetics with propofol showed inconsistent results, preventing the identification of a clear trend in CD4+, CD8+, and Treg counts, as well as in the CD4+/CD8+ ratio.

T lymphocytes hold a prominent role in the immune response, and fluctuations in the numbers of their various subgroups reflect alterations in cell-mediated immunity. The CD3 marker is expressed on the surface of mature T cells, assisting the recognition of Major Histocompatibility Complex (MHC) antigens on antigen-presenting cells by the T-Cell Receptor (TCR) [20]. Consequently, CD3+ cells, which represent the total number of T lymphocytes, including CD4+ and CD8+, reflect the potential for an effective immune response, and any decrease in their number could be indicative of immune dysregulation [21]. CD4+ lymphocytes assume multiple functions in the cellular immune response by secreting IL-2, a prominent growth factor for T-lymphocytes, and through activation of CD8+ cytotoxic lymphocytes. CD8+ T lymphocytes and NK cells are key components of the cellular immune response against tumor cells [20]. CD8+ T cells recognize tumor-associated antigens presented on MHC class I molecules on the surface of infected or transformed cells and eliminate them through the release of perforin and granzymes or via the Fas-FasL signaling pathway. Once activated, CD8+ T cells can induce apoptosis in tumor cells, thereby limiting malignant progression. In contrast, NK cells target cells with reduced MHC-I expression—a common immune evasion strategy of tumor cells. This mechanism enables NK cells to eliminate abnormal cells that escape CD8+ T cell surveillance [20]. NK cells also utilize perforin- and granzyme-mediated cytotoxicity and possess alternative death receptor pathways to induce apoptosis. Together, these immune cells are critical in suppressing tumor growth and preventing metastasis [20]. Furthermore, CD4+ lymphocytes have direct cytotoxic action and coordinate the activity of innate immunity cells, such as macrophages [22]. In patients with HIV (Human Immunodeficiency Virus), the low number of CD4+ lymphocytes and the decreased CD4+/CD8+ ratio have been correlated with increased risk of lung cancer as a result of the deficient immune response [23]. Elevated numbers of circulating tumor cells in lung cancer patients have been linked with a higher likelihood of metastasis [24]. Ye et al. illustrated that a decrease in the number of CD4+ cells and the CD4+/CD8+ ratio is associated with increased circulating tumor cells [25]. Taking into account the aforementioned, the administration of combined general and epidural anesthesia could attenuate the risk of lung cancer recurrence postoperatively.

CD8+ T lymphocytes represent an important part of the cell-mediated immune response through their ability to recognize and eliminate cancer cells selectively [20,26]. It has been argued that CD8+ cell count in peripheral blood could represent an independent prognostic factor for lung cancer [27]. The current meta-analysis did not illustrate superiority of either anesthetic technique with respect to the number of CD8+ T lymphocytes postoperatively. NK cells comprise part of the innate immunity, with similar cytotoxic activity to CD8+ cells without expressing the CD3 marker and the TCR [28]. Likewise, decreased NK cell counts have been linked to a higher number of tumor circulating cells [25]. Quantitative analysis revealed that the addition of epidural anesthesia to general anesthesia led to an increased number of NK cells one day postoperatively, however, without achieving statistical significance.

It has been hypothesized that a different subgroup of Τ cell lymphocytes, Tregs, assumes a role in cancer pathogenesis by inhibiting the activity of other T cell subsets, thus impeding immune responses [29,30]. Peripheral Treg cell counts have been found to be increased in patients with lung cancer [31].

A primary limitation of this systematic review and meta-analysis is the scarcity of available trials. Additionally, the scope of this study is constrained by a paucity of data suitable for statistical analysis. A further limitation is the lack of clinical homogeneity among the included studies regarding lung cancer stage and type, methodology, anesthetic technique, and surgical operation. General anesthesia can be performed either through inhalational agents or as TIVA. It is noteworthy that not all studies employed the same anesthetic agent. This heterogeneity, along with the small number of studies, limits the interpretability of the results and prevents broader extrapolations. Moreover, the subjects of the studies included in the meta-analysis come from China.

Thus, the possibility that these results are only applicable to the Chinese population must be considered. The results of this study must be interpreted with caution as the limited available data prevented the conduct of a predetermined sensitivity analysis and the investigation of publication bias. Larger, carefully designed randomized control studies with homogeneous methodology are required in order to assess with greater precision the impact of anesthetic techniques on the complex immune landscape of the perioperative period. Data pertaining to the choice of anesthetic technique for other types of cancer could be taken into consideration, enabling a more comprehensive comparison.

## 5. Conclusions

The current meta-analysis suggests that the administration of general anesthesia in conjunction with epidural anesthesia is associated with elevated CD3+ and CD4+ T cell counts, as well as an increased CD4+/CD8+ ratio in patients undergoing lung cancer surgery. These findings carry potential implications for anesthesia protocols in thoracic oncology. Primarily, the observed associations indicate that epidural anesthesia may help preserve perioperative immune function, which is particularly important in the context of oncologic surgery. Consequently, anesthesia protocols for lung cancer surgery might consider incorporating regional techniques such as epidural anesthesia not only for pain management, but also for their potential immunomodulatory benefits. However, no definitive conclusions can be drawn regarding the influence of this anesthetic technique on CD8+ T cell and NK cell counts. Additionally, data comparing volatile-based anesthesia with TIVA were insufficient for quantitative analysis and did not reveal any consistent immune-related advantages of either approach. Due to limited data quantity and quality, future randomized controlled trials with standardized methods are urgently required.

## Figures and Tables

**Table 1:** Basic characteristics of included studies.

First Author, Year, Country	Number of Participants (Male/Total)	Lung Cancer Subtype	TNM Classification Group 1	TNM Classification Group 2	TNM Classification Group 3	Type of Surgery	ASA	Age of Participants (Years)
Cui, C. [13] 2022, China	36 Propofol group: 13/18 Sevoflurane group: 12/18	N/A	N/A	N/A	N/A	Lobectomy and lymph node dissection, excluding unilateral pneumonectomy	I–II	N/A
Li, M.-H. [14] 2018, China	64 General anesthesia group: 10/27 General plus EA group: 20/37	Adenocarcinoma	General anesthesia group T1: 16, T2: 9, T3: 1, T4: 1, N0: 24, N1: 0, N2: 3, M0: 27	General anesthesia plus EA group T1: 23, T2: 10, T3: 3, T4: 1, N0: 27, N1: 2, N2: 8, M0: 37	N/A	General anesthesia group Thoracoscopic surgery: pneumonectomy, 0; lobectomy, 26; less resection, 1; General anesthesia plus EA group Thoracoscopic surgery: pneumonectomy, 1; lobectomy, 35; less resection, 1;	General anesthesia group: ASA I: 2, ASA II: 26, ASA III: - General anesthesia plus EA group: ASA I: 0, ASA II: 37, ASA III: -	General anesthesia group: 62.0 ± 10.9 General anesthesia plus EA group: 61.1 ± 8.5
Xu Q. [15] 2017, China	120 General anesthesia group: 43/60 General plus EA group: 44/60	General anesthesia group; squamous cell carcinoma: 18, adenocarcinoma: 31, large cell carcinoma: 5, adenosquamous carcinoma: 6 General plus EA group; squamous cell carcinoma: 20, adenocarcinoma: 30, large cell carcinoma: 5, adenosquamous carcinoma: 5	General anesthesia group Stage II: 27, Stage IIA: 33	General anesthesia plus EA group Stage II: 26 Stage IIA: 34	N/A	Video-assisted thoracoscopic surgery for radical resection: right upper lobectomy, 34; right middle lobectomy, 16; right lower lobectomy, 15; left upper lobectomy, 30; left lower lobectomy, 22; total left lung resection, 3;	N/A	General anesthesia group: 56.2 ± 6.4 General anesthesia plus EA group: 56.1 ± 5.8
Yamaguchi, A. [16] 2021, Japan	64 General anesthesia Desflurane group: 15/20 Sevoflurane group: 15/22 Propofol group: 15/22	Desflurane group; adenocarcinoma: 16, squamous cell carcinoma: 3, metastatic tumor: 1 Sevoflurane group; adenocarcinoma: 15, squamous cell carcinoma: 3, small cell carcinoma: 1, sarcoma: 1, metastatic tumor: 2 Propofol group; adenocarcinoma: 14, squamous cell carcinoma: 7, metastatic tumor: 1	Desflurane group Stage 0: 3, Stage I: 9, Stage II: 4, Stage III: 3, metastatic tumor: 1	Sevoflurane group Stage 0: 2, Stage I: 12, Stage II: 5, Stage III: 1, metastatic tumor: 2	Propofol group: Stage 0: 1, Stage I: 17, Stage II: 1, Stage III: 2. metastatic tumor: 1	Desflurane group: lobectomy, 14; segment resection, 2; partial resection, 4; Sevoflurane group: lobectomy, 16; segment resection, 4; partial resection, 2; Propofol group: lobectomy, 14; segment resection, 7; partial resection, 1;	Desflurane group: ASA I: 0, ASA II: 14, ASA III: 6 Sevoflurane group: ASA I: 2, ASA II: 16, ASA III: 4 Propofol group: ASA I: 2, ASA II: 14, ASA III:	Desflurane group: 69.2 ± 8.9 Sevoflurane group: 69.0 ± 9.0 Propofol group: 68.8 ± 8.9
Yuan, X. [17] 2018, China	246 General anesthesia Sevoflurane group: 85/123 Propofol group: 78/123	N/A	Sevoflurane group Stage I: 12, Stage II: 25, Stage III: 86	Propofol group Stage I: 15, Stage II: 29, Stage III: 79	N/A	Thoracotomy	Sevoflurane group: ASA I: 73, ASA II: 12, ASA III: 8 ASA IV: 30 Propofol group: ASA I: 69 ASA II: 13, ASA III: 9, ASA IV: 32	Sevoflurane group: 43.95 ± 2.01 Propofol group: 46.39 ± 1.85
Zhu, Y. [18] 2017, China	40 General anesthesia group: 11/20 General anesthesia plus EA group: 13/20	N/A	N/A	N/A	N/A	Radical resection of lung cancer, thoracotomy	I–II	General anesthesia group: 59 ± 7 General anesthesia plus EA: 58 ± 6
Chen, J. [19] 2017, China	34 General anesthesia group:13/17 General anesthesia plus EA group:12/17	N/A	N/A	N/A	N/A	Radical resection	I–II	General anesthesia group: 56 ± 6 General anesthesia plus EA: 59 ± 7

Values are presented as the mean ± SD; EA: Epidural anesthesia; TNM: Tumor–Node–Metastasis; ASA: American Society of Anesthesiologists; N/A: Not applicable.

**Table 2:** Anesthesia characteristics of included studies.

First Author, Year, Country	GA Maintenance	Epidural Catheter Characteristics	Duration of the Epidural Catheter	Epidural Medications Intraoperatively	Intraoperative Opioids	Postoperative Analgesia
Cui C. [13] 2022, China	General anesthesia Propofol group Sevoflurane group	N/A	N/A	N/A	Fentanyl 1 μg/kg (when heart rate or blood pressure was higher than 20% of the basic values)	N/A
Li, M.-H. [14] 2018, China	General anesthesia group: propofol General anesthesia plus EA group: epidural ropivacaine, IV propofol	The epidural catheter was placed at the T6–T8 level preoperatively	N/A	0.5% ropivacaine (by continuous infusion at a rate of 4–6 mL/h),	iv sulfentanil/remifentanyl	General anesthesia group: PCIA with 0.5 mg/mL morphine General anesthesia with EA group: PCEA with 0.12% ropivacaine and 0.5 lg/mL sufentanil
Xu Q. [15] 2017, China	General anesthesia group: IV vecuronium General anesthesia plus EA group: epidural ropivacaine, IV vecuronium	The epidural catheter was placed at the T7–T8 level	N/A	0.375% ropivacaine	5 µg/kg fentanyl	N/A
Yamaguchi, A. [16] 2021, Japan	General anesthesia desflurane group sevoflurane group propofol group	The epidural catheter was placed at the T5–T7 level	N/A	4 mL 0.25% levobupivacaine, 50 µg fentanyl, and 1–2 mg morphine, followed by continuous epidural infusion of levobupivacaine (3 mL/h) and morphine (2–3 mg/day)	0.1–0.25 µg/kg/min ramifentanil according to blood pressure and heart rate	Levobupivacaine (3 mL/h) and morphine (2–3 mg/day) via the epidural catheter
Yuan, X. [17] 2018, China	General anesthesia sevoflurane group propofol group	N/A	N/A	N/A	0.1–0.2 µg/kg/min remifentanil	N/A
Zhu, Y. [18] 2017, China	General anesthesia group: propofol General anesthesia plus EA group: rovipacaine epidural, propofol IV	The epidural catheter was placed at the T7–T8 level	N/A	A total of 0.375% ropivacaine (3–5 mL) was administered epidurally every 30 min during surgery, 30 min before the end of surgery, additional ropivacaine was administered	General anesthesia group: 4–6 ng/mL remifentanil (to control blood pressure and heart rate below 1.2 times the baseline), sulfentanil loading dose (5 mL; 0.1 μg/kg) followed by infusion 2 mL/h, sulfentanil through PCA (containing 3 μg/kg sufentanil/150 mL, 2 mL bolus at 15 min) General anesthesia plus EA group: ramifentanil	Epidural rovipacaine and sulfentanil through PCA pump [3 μg/kg sufentanil and 0.2% ropivacaine (300 mL)]- bolus 4 mL at 15 min
Chen J. [19] 2017, China	General anesthesia group: propofol General anesthesia plus EA group: rovipacaine epidural, propofol IV	The epidural catheter was placed at the T7–T8 level	N/A	Ropivacaine 0.375% and 3–5 mL before surgery, repeated every 30 min for 1–2 times during the operation, around 30 min before finishing surgery, ropivacaine was added in the epidural line for the last time as load capacity, analgesia pump was connected to epidural line	Remifentanyl through controlled infusion (target concentration at 3–6 μg/mL)	Epidural sufentanyl 3 μg/kg, ropivacaine 0.2%, flow rate 4 mL/h (dose 300 mL)

IV: intravenous; μg: microgram; ng: nanogram; kg: kilogram; mL: milliliter; h: hour; min: minute; T: thorax; EA: epidural anesthesia; PCA: patient-controlled analgesia; PCIA: patient-controlled intravenous analgesia; PCEA: patient-controlled epidural analgesia; N/A: not applicable.

**Table 3:** Risk of bias assessment for primary outcome with Cochrane RoB tool 2.0.

First Author, Year, Country	Randomisation Process	Deviation from Intended Interventions	Missing Outcome Data	Measurement of the Outcome	Selection of the Reported Result	Overall Bias
Cui C. [13] 2022, China	Some concerns	Some concerns	Low	Low	Low	Some concerns
Li, M.-H. [14] 2018, China	Low	Some concerns	Low	Low	Low	Some concerns
Χu, Q. [15] 2017, China	Some concerns	Some concerns	Low	Low	Low	Some concerns
Yamaguchi, A. [16] 2021, Japan	Some concerns	Some concerns	Low	Low	Low	Some concerns
Yuan, X. [17] 2018, China	Some concerns	Low	Low	Low	Low	Some concerns
Zhu, Y. [18] 2017, China	Some concerns	Some concerns	Low	Low	Low	Some concerns
Chen, J. [19] 2017, China	Some concerns	Some concerns	Low	Low	Low	Some concerns

## Data Availability

No new data were created or analyzed in this study.

## Supplementary Materials

The following supporting information can be downloaded at https://www.mdpi.com/article/10.3390/medicina61071263/s1, A Prisma 2020 27-item Checklist and Prisma 2020 Abstract checklist are provided [32].

## Abbreviations

The following abbreviations are used in this manuscript:

RCT	Randomized Controlled Trial
HPA axis	Hypothalamic–Pituitary–Adrenal axis
SNS	Sympathetic Nervous System
NK cells	Natural Killer cells
NKT cells	Natural Killer T cells
Th1	T helper 1
Th2	T helper 2
Th17	T helper 17
Treg cells	T regulatory cells
TIVA	Total Intravenous Anesthesia
ASA	American Association of Anesthesiologists
RoB	Risk of Bias
SMD	Standardized Mean Difference
Cl	Confidence Interval
RevMan	Review Manager
TNM	Tumor–Node–Metastasis
TNF-a	Tumor Necrosis Factor-a
IL	Interleukin
N/A	Not Available
TCR	T-cell receptor
MHC	Major Histocompatibility Complex
